# Relationship Between Perilesional Skin Condition and Survival in Terminally Ill Patients with Pressure Ulcers

**DOI:** 10.3390/medicina61010147

**Published:** 2025-01-17

**Authors:** María Isabel Pastor-Orduña, Federico Palomar-Llatas, David Palomar-Albert, María Teresa Murillo-Llorente, Ignacio Ventura, Francisco Tomás-Aguirre, Marcelino Pérez-Bermejo

**Affiliations:** 1 Doctoral School, Catholic University of Valencia San Vicente Mártir, 46001 Valencia, Spain; pastor_marord@gva.es; 2 Chair of Integrity and Skin Care, Integrity and Skin Care Research Group, Catholic University of Valencia San Vicente Mártir, 46001 Valencia, Spain; federico.palomar@ucv.es (F.P.-L.); david.palomar@ucv.es (D.P.-A.); 3 SONEV Research Group, School of Medicine and Health Sciences, Catholic University of Valencia San Vicente Mártir, 46001 Valencia, Spain; mt.murillo@ucv.es (M.T.M.-L.); paco.tomas@ucv.es (F.T.-A.); 4 Molecular and Mitochondrial Medicine Research Group, School of Medicine and Health Sciences, Catholic University of Valencia San Vicente Mártir, 46001 Valencia, Spain; ignacio.ventura@ucv.es

**Keywords:** pressure ulcers, palliative care, survival, epithelialization, function, albumin

## Abstract

*Background and Objectives:* In the context of palliative care, the aim is to alleviate suffering and improve quality of life, with particular attention to PUs, which have a significant impact on quality of life and survival. This study examines the relationship between perilesional skin condition and survival in terminally ill patients with pressure ulcers (PUs). *Materials and Methods:* A descriptive and observational study was conducted in two hospitals in Valencia with a sample of 100 terminally ill patients. Sociodemographic, clinical and PPU-specific variables were assessed using validated scales such as FEDPALLA-II and the Barthel Index. *Results:* Although it is a study of an observational nature, which may preclude establishing causality, the results showed that functional capacity, perilesional tissue epithelialization, and albumin levels were significant predictors of survival, while the number and location of PUs had no direct impact. Perilesional tissue epithelialization was highlighted as a critical indicator reflecting the systemic stability of the patient. *Conclusions:* The study highlights the importance of a comprehensive approach to palliative care that addresses both the local aspects of the lesions and the patient’s systemic and functional status. These findings support the implementation of therapeutic interventions based on a structured perilesional tissue assessment to improve quality of life and prolong survival in terminally ill patients. In addition, a positive correlation was found between Barthel Score and survival, suggesting that patients with greater functional independence have a longer life expectancy. On the other hand, the negative correlation between total lymphocyte count and survival suggests that lymphocytopenia may be a marker of adaptive immunosuppression. Perilesional tissue epithelialization, overall functionality and serum albumin levels are key factors in predicting survival, highlighting the need for a comprehensive palliative care approach to optimize quality of life and prolong survival in terminally ill patients with PUs.

## 1. Introduction

Terminal, advanced, progressive, and incurable illnesses are characterized by intense and multifactorial symptoms, a life expectancy of less than six months, and a high emotional impact on the patient, family, and healthcare team [1].

In this context, palliative care (PC) aims to alleviate physical and emotional suffering, prioritizing quality of life and comprehensive care, including aspects such as the management of pressure ulcers when their cure is not possible, attention to suffering when death is imminent, and helping to die in peace, as also proclaimed by the Declaration of the Parliamentary Assembly of the Council of Europe on the Rights of the Terminally Ill and Dying [2,3]. According to the WHO, chronic diseases, including cancer and cardiovascular diseases, are responsible for 60% of premature deaths worldwide [4].

Complications, whether oncologic or not, that occur in any disease in its terminal phase usually have common symptoms that should be evaluated according to their specific characteristics, such as frequency, severity, conditions of presentation and persistence over time. It is also necessary to evaluate the impact on the functional situation and on the psychological, spiritual, and family spheres [5].

Among the most common complications in terminally ill patients are pressure ulcers (PUs), which not only compromise skin integrity but are also associated with a significant impact on quality of life and survival [6]. PUs represent a major health and social problem today. According to the definition of the National Group for the Study and Advice on Pressure Ulcers and Chronic Wounds (GNEAUPP) in 2014, in its second revision, a pressure injury is of ischemic origin, located in the skin and underlying tissues with loss of cutaneous substance and produced by prolonged pressure [7,8], it was subsequently redefined by the National Pressure Ulcer Advisory Panel (NPUAP) and the European Pressure Ulcer Advisory Panel (EPUAP) as a localized injury to the skin or underlying tissue, usually over a bony prominence, as a result of pressure or pressure combined with shear [9].

The prevention and treatment of PUs in bedridden and chronically ill patients is directly related to the quality of care provided, as it is one of the basic functions of the nursing profession [10]. PUs affect a large proportion of the population, especially the elderly and patients with reduced mobility, and are of great economic and social importance, as well as reducing the quality of life of patients and even their families [7,8].

The term “Kennedy Terminal Ulcer” (KTU) was coined in 1983 by Karen Lou Kenne-dy, who, while working in a skin care team, observed that some people suffering from a particular type of PU died approximately two weeks after onset. KTUs are lesions characterized by their rapid progression and association with increased mortality in patients with advanced disease. Their relevance lies in the fact that they reflect, at a glance and in a clinically unambiguous manner, the patient’s systemic vulnerability and its potential impact on survival outcomes. Although these lesions have been studied primarily from a clinical perspective, there is a gap in the literature regarding how perilesional skin integrity may influence clinical outcomes in terminally ill patients [11,12,13,14,15,16].

In the terminal phase, the skin reflects what is happening in the organism. Several authors state that as people approach the dying process, their internal organs begin to decrease in function and suffer from multiorgan failure, a phase in which all organs begin to slow down and stop functioning efficiently [6,17,18]. Almost all people in palliative care are susceptible to the development of PUs, and therefore, risk assessment and prevention strategies are strongly recommended as an essential component of palliative care to maintain the patient’s quality of life [19].

Within these strategies, evaluation of the perilesional skin plays a critical role in the prevention and treatment of these lesions. Unlike ulcerated skin, healthy perilesional skin has a greater capacity to metabolize and perform its functions as a protective barrier. The integrity of this skin is essential to the prevention and management of the lesion area, as its integrity is an important protective factor [20]. This assessment includes an evaluation of factors that influence ulcer development, such as oxygenation and blood flow, which are critical for wound healing. Indicators such as blood pressure, flushing, and immediate capillary refill are clinically assessable and associated with wound bed development. In addition, the protein content and pH of the skin are important in assessing the immune capacity of the new tissue and the efficacy of anti-infective treatments [21].

Given that the skin reflects critical systemic changes in the terminal phase, this study aims to explore how the state of the perilesional tissue may influence the survival of patients with PU and provide evidence for more effective palliative interventions.

## 2. Materials and Methods

### 2.1. Settings and Participants

We conducted a descriptive, observational, and post-prospective study using an accidental sample of patients in palliative or terminal care who had PUs on admission or developed during their stay. The centers where the study was carried out were the Consorcio del Hospital General Universitario de Valencia (CHGUV), which is a tertiary-level hospital with a specialized palliative care unit designed to care for patients with advanced and terminal chronic pathologies, and the Hospital de Atención a Crónicos y Larga Estancia (HACLE) Dr. Moliner, located in Serra (Valencia), which specializes in the prolonged care of patients with advanced chronic diseases and continuous care needs.

Patients were consecutively recruited if they met the following inclusion criteria: they were adults, had no cognitive impairment, understood Spanish, had advanced and life-threatening illness in a late palliative phase, were eligible for inclusion in a palliative or end-of-life care program, and had PDUs on admission or developed during their stay. In addition, eligible patients had to be personally aware of being in a palliative phase and receiving palliative care. Data collection took place between 2020 and 2024, with partial interruptions due to the COVID pandemic. To ensure the consistency and reliability of data collection, a single trained nurse was assigned to assess all patients using the validated tools. This approach minimized potential measurement discrepancies and ensured a high degree of uniformity in the data collected, thereby reducing bias related to interobserver variability. We chose to use consecutive sampling because it provided feasible access to the study population. However, a post-hoc power analysis will be performed based on the results of the multiple linear regression. The study design required regular visits to the hospitals three times a week to identify new inpatients who met the inclusion criteria and to monitor the clinical status of those already recruited. All patients in the sample died during hospitalization.

### 2.2. Variables

Sociodemographic and clinical variables were collected (age, sex, admission and associated pathology, number of PUs, age of lesion, anatomical location, category or stage, length, width and depth of PU, characteristics of ulcer tissue, characteristics of perilesional skin, type and amount of exudate, signs of infection, days since PU appearance). Pressure ulcers were classified according to the European Pressure Ulcer Advisory Group [22].

In addition, the following validated scales were used to achieve the objectives of the study:

#### 2.2.1. FEDPALLA-II Index

The FEDPALLA-II scale allows for the evaluation of the perilesional lesion according to five dimensions or parameters: hydration, eczematization, vascularization, margins, and dermal deposits. Its application makes it possible to improve the clinical efficacy of PUs, to select an appropriate topical treatment and to document the evolution of the epithelialization process [23]. It is scored from 5 to 25 points and defines four reference grades for the prognosis of epithelialization: Grade I, from 21 to 25 points, indicating a very good prognosis, Grade II, from 16 to 20 points, good prognosis, Grade III, from 11 to 15 points, poor prognosis and Grade IV, from 05 to 10 points, very poor prognosis.

#### 2.2.2. Barthel Index

The Barthel Index is a scale that assesses the patient’s level of independence [24] through the ability to perform basic activities of daily living (BADLs) such as eating, washing, dressing, bowel movements, urination, toileting, transferring, walking, and climbing and descending stairs.

This scale is very useful for its validity, sensitivity and reliability in describing the functional status of older adults [24,25,26,27] and is considered the most useful scale for assessing ADLs because of its characteristics, low cost and potential usefulness for monitoring patient progress. It is scored from 0 to 100 and defines four categories of dependency: total dependency, less than 20; severe, between 20 and 40; moderate, between 40 and 55; and mild, greater than 60.

### 2.3. Statistical Analysis

Data were summarised in tables and analyzed descriptively and inferentially using SPSS 25.0 (SPSS Inc., Chicago, IL, USA). For descriptive data, central tendency and dispersion statistics are used for continuous variables. Qualitative variables are expressed as case and per-case values. The normality of distributions was analyzed using the Shapiro–Wilk test. The Kruskal–Wallis test was used to compare values of continuous variables, and the Pearson test was used to determine correlations between them. After adjusting potential confounding variables, multivariate linear regression models were used to investigate factors predictive of survival, with stepwise selection of independent variables. Assumptions of the resulting model were tested using the Durbin–Watson statistic to analyze independence in residuals and tolerance values, and the Variance Inflation Factor (VIF) value for multicollinearity between predictor variables. A significance level of 0.05 was used for all tests.

### 2.4. Ethical Approval and Informed Consent

The study was conducted in accordance with the tenets of the Declaration of Helsinki. Approval was obtained from the Ethics Committee CEIm Consorcio Hospital General Universitario de Valencia, dated 4 October 2018, and informed consent was obtained from all participants.

## 3. Results

The final sample analyzed was 100 patients. A total of 45 were men (45%) and 55 were women (55%), reflecting the parity of participants (z-test; *p* = 0.157). The mean age was 76.2 years (SD = 12.1) with a range of 39 to 96 years. Fifty per cent of participants were over 79 years old. Table 1 describes the sociodemographic characteristics of the sample.

We found no statistically significant differences between the number of PUs and survival days after infection (Kruskal–Wallis test: *p* = 0.399). We also found no statistically significant differences between the number of ulcers and the location of the lesion (Kruskal–Wallis test: *p* = 0.394). Although this is a valid result, and despite the fact that the number of ulcers does not affect survival, the comprehensive therapeutic intervention may buffer these differences. This finding suggests that in this terminal population, the number of pressure ulcers does not have a direct significant impact on survival, possibly due to intensified palliative care that mitigates the effect of local variables.

**Figure 1:**
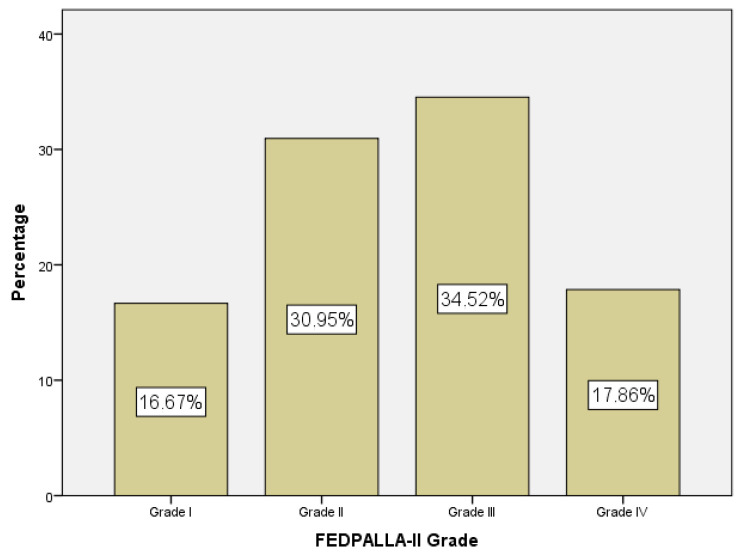
Sample distribution according to the FEDPALLA-II Grade.

**Figure 2:**
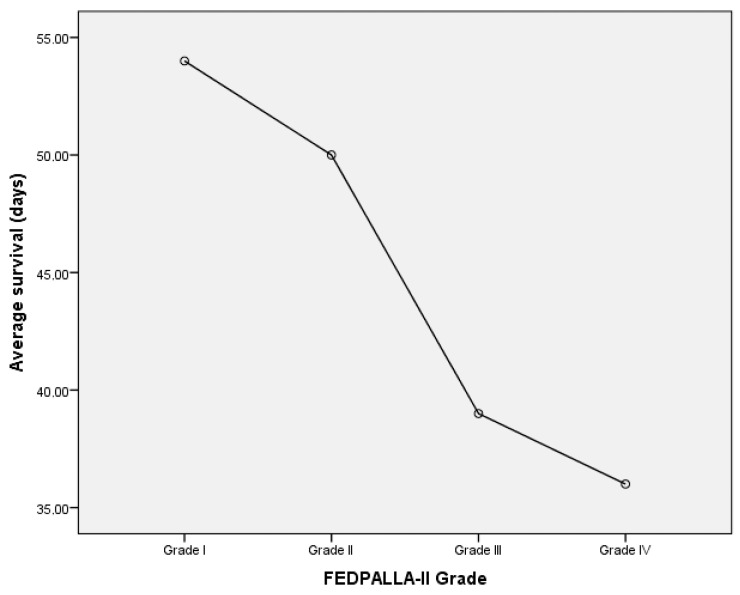
Survival of the sample according to FEDPALLA-II Grade.

With regard to the FEDPALLA-II index, Figure 1 shows the distribution of the sample and Figure 2 shows the mean survival, both according to grade. We found that survival increases with a better score on this scale, which seems to show that it is a robust tool for evaluating the state of the perilesional skin, positioning it as a key indicator in the management of palliative patients with ulcers.

We found a strong, statistically significant positive correlation between Barthel Score and survival (r = 0.692; *p* < 0.001), suggesting that patients with greater functional independence have a longer life expectancy. On the other hand, we found a negative correlation between total lymphocyte count and survival (r = −0.449; *p* = 0.021), suggesting that lymphocytopenia could be a marker of adaptive immunosuppression, which in turn would be associated with longer survival by reducing complications associated with an uncontrolled inflammatory response.

Table 2 shows the results of the multiple regression analysis examining the predictors of survival. Although all variables were considered as possible predictors, only those that were statistically significant are shown in the final model. The overall model is statistically significant (multiple correlation coefficient = 0.852), indicating a strong relationship between the variables included in the analysis. Approximately 72% of the variance in survival can be explained by the linear combination of Barthel score, epithelialization, and albumin. The F statistic (85,679) testing the overall regression relationship is highly significant (*p* < 0.001), indicating a significant association between the set of predictor variables and patient survival.

We can see that the Barthel score, epithelialization, and albumin all have a very significant effect on survival. The higher the value of these three predictors, the longer the expected survival time.

Assumption checks revealed a Durbin–Watson statistic of 1.719, which supports the assumption of independence in the residuals. In addition, the tolerance values (0.506, 0.560 and 0.351) and the VIF value (1.978, 1.984, and 2.851) indicate that there is no multicollinearity between the predictor variables. These results highlight the importance of a comprehensive approach that includes both local (epithelialization) and systemic (functionality and nutrition) factors in predicting survival in terminally ill patients with pressure ulcers, reinforcing the need to systematically monitor and manage these aspects in palliative care.

## 4. Discussion

This study analyzed 100 terminally ill patients with PUs in the last days of life and identified important factors associated with survival. We analyzed the relationship between perilesional skin condition and survival in terminally ill patients with pressure ulcers. Our results suggest that factors such as functionality, perilesional tissue epithelialization and albumin levels are significant predictors of survival in this population. In contrast, the number and location of ulcers had no direct effect on remaining life expectancy, highlighting the importance of comprehensive palliative care.

These results highlight the impact of functionality, perilesional tissue status and nutritional markers on survival in terminally ill patients with PUs and provide evidence to inform therapeutic strategies in the palliative setting. Perilesional tissue epithelialization is one of the most significant predictors of survival in our study, highlighting the importance of perilesional tissue integrity not only in terms of local wound management but also as a reflection of the patient’s systemic stability. Improved epithelialization may indicate a controlled inflammatory response and improved tissue perfusion, which are associated with reduced systemic complications and increased life expectancy [28].

The lack of statistically significant differences between survival and the number or location of PUs may be explained by the complexity of systemic factors that predominate in terminally ill patients. In this population, survival is profoundly influenced by the patient’s general condition, comorbidities, and pathophysiological processes of the last days, which overshadow the local impact of PUs [29]. Although the number and location of PUs reflect a local tissue status, these factors do not seem to have a determinant weight on survival, which is more related to systemic conditions such as multiorgan failure, systemic inflammatory response, and the global functionality of the patient [30]. Although PUs are a common and debilitating complication in terminally ill patients, our results suggest that the number and location of ulcers do not directly affect survival in this setting. This may be due to the implementation of intensive palliative care that minimizes the impact of these lesions by prioritizing pain control, infection prevention and intensive hydration. Consequently, the number and location of ulcers may be less relevant in patients whose survival is determined more by their overall functional status and systemic conditions, such as multiorgan failure [30].

Intensive palliative management strategies could help to reduce the differences in survival associated with the number and location of PUs. In terminally ill patients, the focus is on pain relief and infection prevention through the use of appropriate dressings, intensive hydration and conservative debridement, thus limiting tissue damage and homogenizing the impact of PUs on the overall clinical course, thus minimizing the observed differences in survival [31]. On the other hand, factors such as functional status or systemic markers such as albumin have a much greater impact on the life expectancy of these patients, suggesting that the role of PUs as independent predictors of survival is limited in this specific context [32,33].

The Barthel Score has a significant impact on the survival of terminally ill patients by assessing an individual’s functional capacity and level of dependence on basic activities of daily living [34]. A higher score reflects greater independence, suggesting greater physiological reserve and overall ability to cope with the systemic stress associated with terminal illness. This residual functionality may be related to a reduced impact of comorbidities and a greater tolerance to associated complications such as infection, development of pressure injuries or worsening of pressure injuries. In addition, patients with less dependency are often better able to mobilize, which improves tissue perfusion and reduces the risk of accelerated systemic deterioration, including the prevention of metabolic and cardiovascular complications that are common in the terminal setting [34,35]. The Barthel Index is also associated with improved access to specialist care, as more independent patients are able to actively participate in their own care, facilitating the implementation of therapeutic interventions such as postural changes, adequate hydration, and more effective wound management. In contrast, patients with greater functional dependence tend to have higher rates of immobilization and associated complications, contributing to progressive systemic deterioration and poorer survival [36]. This marker therefore integrates both physical and functional aspects of the patient’s condition, which explains its influence on prognosis and its predictive value in survival models [33,34,35,36,37].

The correct assessment and treatment of the perilesional skin, especially using tools such as FEDPALLA-II, is crucial in terminally ill patients with PPU, not only to improve quality of life but also to prolong survival. According to Palomar Llatas et al. [23], FEDPALLA-II stands out as a comprehensive tool that allows the assessment of key parameters of the perilesional tissue, such as hydration, vascularization and epithelialization, which not only influence local healing but also modulate systemic processes associated with inflammation and progressive patient deterioration [23]. In the present study, epithelialization emerged as a significant predictor of survival, highlighting its ability to identify critical aspects of clinical management in a highly vulnerable population.

The impact of FEDPALLA-II as a predictive marker is consistent with previous findings linking optimization of perilesional tissue with a reduction in systemic complications. Palomar Llatas et al. [23] also pointed out that perilesional tissue deterioration can act as a perpetual source of inflammation, exacerbating metabolic stress and contributing to multiorgan failure, a major cause of mortality in terminally ill patients. In this context, an elevated FEDPALLA-II score reflects not only local improvement but also stabilization of the inflammatory cascade, leading to increased life expectancy [38,39]. The use of FEDPALLA-II also provides a structured and reproducible approach to PU management, allowing objective monitoring of the evolution of therapeutic interventions, facilitating evidence-based clinical decisions. In the present study, epithelialization shows its ability to predict survival in a highly vulnerable population. This finding is consistent with work highlighting the beneficial effects of interventions aimed at improving the wound bed and perilesional skin in critically ill patients [40,41].

Compared to other tools, it offers a more comprehensive approach by incorporating perilesional parameters that have a direct impact on therapeutic management. Studies such as those by Sibbald et al. [40] and Guo et al. [41] support that systematic assessment of perilesional tissue significantly improves clinical outcomes by allowing more precise and targeted interventions, which is consistent with our results showing a significant relationship between epithelialization and survival time.

Furthermore, an assessment of epithelialization correlates with a comprehensive therapeutic approach in which advanced perilesional skin management plays a critical role. Interventions such as intensive hydration, use of bioactive dressings and reduction of local bacterial load not only improve the parameters assessed by FEDPALLA-II, but also mitigate systemic complications such as sepsis and excessive release of inflammatory mediators. This approach is particularly relevant in terminally ill patients where systemic responsiveness is limited and local interventions become disproportionately important for clinical stabilization [31,42].

The FEDPALLA-II scale is not only a clinical marker, but also an educational tool that allows multidisciplinary teams to identify critical areas for intervention [23]. Its use promotes evidence-based care, optimizes resources and prioritizes interventions that have a direct impact on the survival and quality of life of the terminally ill, confirming that it is an indispensable tool for the assessment and management of perilesional tissue in this population and consolidating its role in advanced clinical practice [31,38,39,40,41,42,43].

The significant negative correlation observed between serum albumin levels and epithelialization in terminally ill patients during the last days of life poses a dilemma of interpretation, as it seems contradictory a priori. This statistical finding, supported by a strong significance, could reflect a compensatory response to intensive local care in patients with low albumin. However, this phenomenon could be influenced by both the direct effects of systemic inflammation and the benefits of local interventions. Future studies that evaluate these factors simultaneously may help to better differentiate their relative effects and interactions. Serum albumin is widely recognized as a systemic marker that reflects not only the nutritional status of the patient but also the level of chronic inflammation and metabolic capacity [44]. In terminally ill patients, albumin levels are profoundly affected by processes such as hypercatabolism, chronic systemic inflammatory response, and liver dysfunction secondary to multiorgan failure [45]. These conditions, characteristic of the terminal phase of life, significantly reduce hepatic albumin synthesis, regardless of nutritional or metabolic support. Consequently, albumin in this population becomes a marker of systemic deterioration rather than of the regenerative or healing capacity of the organism [46,47].

The FEDPALLA-II score focuses on local assessment of perilesional skin condition, taking into account factors such as hydration, inflammation, vascularization, and superficial deposits. Unlike albumin, this indicator is more influenced by specific skin care and does not necessarily reflect the patient’s systemic status. In palliative care, it is common to prioritize intensive skin interventions to relieve local symptoms, improve comfort, and prevent complications such as infection or severe ulceration [48].

The significant negative correlation between these two parameters could be explained by a clinical compensation phenomenon. Patients with lower albumin levels, reflecting a worse systemic condition, might receive more intensive palliative skin care, which would contribute to an improved epithelialization score. In contrast, those with higher albumin levels may not require such extensive local care, resulting in slightly lower scores on this scale. This dynamic reinforces the hypothesis that both markers are modulated by independent processes that may not evolve synchronously in the terminal context [43].

In terminal patients, it is common to observe a time lag between systemic and local changes. While albumin levels may deteriorate rapidly due to the progression of the terminal disease, perilesional skin conditions may have previously improved as a result of continuous and specific care. This physiological decoupling between systemic and local status may contribute to the observed negative correlation, where parameters do not reflect the same clinical status of the patient at a given time [38,41].

The effects of systemic comorbidities in diseases such as advanced cancer, congestive heart failure and chronic kidney disease significantly affect albumin levels but do not always have a direct correlation with perilesional skin condition, especially when the latter is under intensive care, reinforcing the idea that albumin and epithelialization are influenced by distinct but co-existing pathophysiological processes in this population [49]. The significant negative correlation between serum albumin and FEDPALLA-II score in terminally ill patients reflects the complex interplay between systemic deterioration and intensive palliative skin care. Although systemic conditions such as multiorgan failure have a significant impact on survival in terminally ill patients, a more detailed investigation of how specific diseases such as cancer, heart failure or renal disease interact with PU treatment may provide more precise guidance for therapeutic strategies. This approach would allow further personalization of care and prioritization of specific interventions according to individual patient needs.

Future research should explore markers of systemic inflammation such as IL-6 or TNF-alpha to complement the analysis of perilesional tissue and evaluate the influence of specific comorbidities such as diabetes or renal failure. Similarly, future studies could address the role of polypharmacy, psychosocial factors such as caregiver support, and specific nutritional interventions to improve albumin levels, the integration of technological advances such as artificial intelligence-based skin assessment tools, and consideration of the economic implications of perilesional care.

Our findings highlight the importance of a comprehensive assessment of terminally ill patients that includes not only local pressure ulcer management but also their functional status, inflammation management and nutritional markers. The combination of tools such as the Barthel Score and the FEDPALLA-II scale could optimize the identification of patients at higher risk of deterioration and help to personalize palliative treatments, thereby improving their quality of life and potentially prolonging their survival.

Effective management of PUs in terminally ill patients requires a multidisciplinary approach that combines the skills and knowledge of different professionals. This approach is essential not only for prevention but also for comprehensive treatment and management of skin lesions, which would directly contribute to improving patients’ quality of life. The integration of physicians, nurses, assistants, orderlies, nutritionists, and physiotherapists allows for personalized care adapted to the specific needs of each patient, addressing both risk factors and pharmacologic and non-pharmacologic therapeutic strategies [50]. Interprofessional communication is a key pillar to coordinate effective care processes, identify risk factors early, and optimize PU treatment. The inclusion of a dietitian in multidisciplinary teams has proven to be an effective strategy to address malnutrition, a condition closely associated with increased risk of complications and mortality in terminally ill patients. Nutritional management not only improves healing processes but also contributes to reducing associated morbidity and mortality, as indicated by the recommendations of the Ethics Working Group of the Spanish Society of Clinical Nutrition and Metabolism (SENPE) [51].

In addition, wound units, which exist in many healthcare centers, have been a significant advance in the multidisciplinary approach to PUs. These specialized units, staffed by professionals trained in the management of chronic and complex wounds, have significantly reduced associated complications by providing comprehensive care, specialized treatment, and counseling to patients with compromised skin integrity [52]. These strategies not only optimize patient care but also reduce the burden of care by implementing evidence-based protocols. The combination of these interventions in a structured and collaborative care model significantly improves clinical outcomes while reducing the incidence of complications.

### Limitations

This study has some limitations, such as the relatively small sample size and its observational nature, which precludes establishing causality. In addition, the sample was recruited by non-probability sampling, and non-representativeness is a common problem when non-probability sampling methods are used. However, we believe that the selection of the centers largely mitigates this problem, as they are specialized referral units for the treatment of terminally ill patients. Nevertheless, as indicated in the methodology, a post-hoc statistical power analysis for multiple linear regression with three predictors was performed using G*Power software v.3.1.9.6 [53], a flexible statistical power analysis program for social, behavioral, and biomedical sciences, and a power of 96% was obtained.

Although the FEDPALLA-II scale is effective in assessing perilesional skin integrity, its focus is limited to local parameters, which may be considered a limitation in capturing the influence of dynamic systemic changes in the evolution of the terminally ill patient. However, we believe that our study mitigates this limitation by including key systemic parameters, such as albumin and lymphocyte levels, which allow a more comprehensive assessment of the patient’s clinical status. Although acute phase reactants such as PCR were not measured, the included parameters provide relevant information on nutrition, immune status, and body responsiveness, which enhances the understanding of the underlying pathophysiological processes. These data showed significant correlations with ulcer development and survival, underscoring the utility of a multidimensional approach combining local and systemic assessment to optimize treatment strategies and personalize care in palliative patients with pressure ulcers.

On the other hand, disruptions related to the COVID-19 pandemic may have affected the consistency of the data collected as the saturation of healthcare systems during this period blurred the definition of “palliative patient”, which affected recruitment capacity. Many patients who would normally have been eligible for this type of research could not be included due to access restrictions, changes in care priorities, and an increase in deaths before or during hospitalization. It would be valuable to conduct multicenter, prospective studies with larger probabilistic samples to confirm these findings and to further explore the mechanisms underlying the relationship between perilesional care and survival in terminally ill patients, as well as to include comparative assessments between different observers to further validate the reproducibility of the measurements.

## 5. Conclusions

The results of this study highlight the importance of perilesional tissue epithelialization as a significant predictor of survival in terminally ill patients with pressure ulcers, reflecting the combined effect of effective local treatment and modulation of associated systemic processes. In addition, general functioning and serum albumin levels were also shown to be important predictors of survival. This highlights the importance of a comprehensive approach that considers both the local aspects of the lesions and the systemic and functional status of the terminally ill patient. Although the number and location of ulcers did not show a direct influence on survival, intensive palliative care appears to mitigate these differences, highlighting the need for early and ongoing management strategies to optimize quality of life and prolong survival. These results strengthen the rationale for implementing therapeutic interventions based on structured perilesional tissue assessment and confirm the role of tools such as FEDPALLA-II in advanced clinical practice in palliative care.

## Figures and Tables

**Table 1:** Sociodemographic and clinical characteristics of patients.

Variable	*n* (%) or Mean (SD) [Range]	*p*-Value *
Gender		
Male	45 (45.0)	
Female	55 (55.0)	
Age	76.2 (12.1) [39–96]	
Admission pathology		
Oncologic	41 (41.0%)	
Non-oncologic	59 (59.0%)	
Associated pathology		
Neurological	46 (46.0%)	
Metabolic	5 (5.0%)	
Renal	3 (3.0%)	
Vascular	4 (4.0%)	
Hepatic	5 (5.0%)	
Infectious	2 (2.0%)	
Cardiac	6 (6.0%)	
Origin of PUs		
Prior to admission	54 (54.0%)	
During hospitalization	46 (46.0%)	
Number of PUs		
1	51 (51.0%)	
2	24 (24.0%)	
3	11 (11.0%)	
>3	14 (14.0%)	
Higher stage PUs		
Grade I	7 (7.0%)	
Grade II	14 (14.0%)	
Grade IIII	29 (29.0%)	
Grade IV	50 (50.0%)	
Total lymphocytes	5.5 (8.46) [0.5–36.7]	0.399
Hematocrit	35.0 (6.46) [25–50]	
Glucose	111.4 (37.9) [72–300]	
Albumin	2.7 (1.0) [1.7–4.5]	
Survival (days)		
Global	45.9 (22.7) [1–200]	0.394
1 PU	40.0 (13.8) [1–120]	
2 PU	47.5 (21.2) [8–120]	
3 PUS	50.1 (18.3) [4–200]	
>3 PU	58.9 (16.2) [1–120]	
FEDPALLA-II Grade I	54.5 (16.7) [20–120]	
FEDPALLA-II Grade II	50.6 (19.1) [2–200]	
FEDPALLA-II Grade III	38.8 (29.4) [10–79]	
FEDPALLA-II Grade IV	36.9 (16.9) [1–49]	
Lower limb PUs	41.1 (16.5) [2–89]	
Gluteal PU	48.2 (12.2) [4–200]	
Sacrum PU	37.3 (17.6) [1–102]	
Heel PU	34.6 (14.7) [1–80]	
Other PU	55.7 (11.5) [10–120]	

* Kruskal–Wallis Test.

**Table 2:** Summary Table Showing Results of Multiple Regression Analysis.

Criterion	Predictor	*t*	β	*p*-Value
Survival (days)	Barthel score	5.822	0.554	<0.001
	Epithelialization (FEDPALLA-II)	4.149	24.499	<0.001
	Albumin	2.972	5.188	0.004
R	0.852			
Adjusted R square	0.718			
F = 85.679, *p* ≤ 0.001				

## Data Availability

The data presented in this study are available on request from the corresponding author. The data are not publicly available due to privacy or ethical restrictions.
